# Prediction of Poor Outcome Using the Urea to Albumin Ratio in Thoracic Empyema

**DOI:** 10.15388/Amed.2024.31.1.21

**Published:** 2024-02-27

**Authors:** Evgeni Dimitrov, Daniel Valchev, Georgi Minkov, Emil Enchev, Yovcho Yovtchev

**Affiliations:** 1 Clinic of Surgical Diseases, University Hospital “Prof. Dr. Stoyan Kirkovich” Stara Zagora, Bulgaria Department of Surgical Diseases and Anesthesiology, Faculty of Medicine, Trakia University Stara Zagora, Bulgaria; 2 Clinic of Thoracic Surgery, University Hospital “Prof. Dr. Stoyan Kirkovich” Stara Zagora, Bulgaria

**Keywords:** empyema, pleural infection, urea, albumin, urea to albumin ratio, UAR, mortality, empiema, pleuros infekcija, karbamidas, albuminas, karbamido ir albumino santykis, UAR, mirtingumas

## Abstract

**Purpose:**

The prognostic performance of urea-to-albumin ratio (UAR) has been assessed in various pulmonary and nonpulmonary conditions, but never in thoracic empyema. Therefore, our aim was to determine whether this marker has the ability to predict outcome in such patients.

**Methods:**

A single-center retrospective study was conducted in a Clinic of Thoracic Surgery at a University Hospital between January 2021 and October 2023. A total of 84 patients who underwent emergency surgery due to thoracic empyema were involved. Serum levels of urea and albumin at admission were used to calculate UAR. We analyzed area under receiver operating characteristics (AUROC) curves of UAR, systemic inflammatory response syndrome (SIRS) and quick-sequential organ failure assessment (qSOFA), and compared their prognostic performance.

**Results:**

The identified in-hospital mortality was 10.7%. The UAR showed the best ability to prognosticate mortality compared to qSOFA (AUROC = 0.828 vs 0.747) and SIRS (AUROC = 0.828 vs 0.676). We established a sensitivity of 87.5% and specificity of 74.2% at optimal cut-off value UAR > 51.1 for prediction of adverse outcome.

**Conclusion:**

In patients with thoracic empyema urea-to-albumin ratio showed significant prognostic performance and a potential for clinical application as a low cost and widely available predictor of death.

## Abstract (it)

**Tikslas:**

Karbamido ir albumino santykio (UAR) prognozė buvo vertinama įvairių plaučių ir ne plaučių ligų atveju, tačiau niekada nebuvo vertinta esant krūtinės ląstos empiemai. Todėl mūsų tikslas buvo nustatyti, ar šis žymuo gali prognozuoti tokių pacientų ligos baigtį.

**Metodai:**

Nuo 2021 m. sausio mėn. iki 2023 m. spalio mėn.Vilniaus universiteto ligoninės Krūtinės chirurgijos klinikoje buvo atliktas vieno centro prospektyvusis tyrimas. Iš viso tyrime dalyvavo 84 pacientai, kuriems buvo atlikta skubi operacija dėl krūtinės ląstos empiemos. UAR apskaičiuoti buvo naudojamas karbamido ir albumino kiekis kraujo serume, buvęs priėmimo į centrą metu. Analizavome UAR, sisteminio uždegiminio atsako sindromo (SIRS) ir greito organų nepakankamumo vertinimo (qSOFA) ploto pagal imtuvo operacines charakteristikas (AUROC) kreives ir palyginome jų prognostinius rodiklius.

**Rezultatai:**

Nustatytas hospitalinis mirtingumas buvo 10,7 %. UAR parodė geriausią mirtingumo prognozavimo gebėjimą, palyginti su qSOFA (AUROC = 0,828 vs. 0,747) ir SIRS (AUROC = 0,828 vs. 0,676). Nustatėme 87,5 % jautrumą ir 74,2 % specifiškumą, esant optimaliai ribinei vertei UAR > 51,1 nepalankiai baigčiai prognozuoti.

**Išvada:**

Krūtinės ląstos empiema sergantiems pacientams urėjos ir albumino santykis pasižymėjo reikšmingu prognostiniu efektyvumu ir klinikinio pritaikymo galimybėmis kaip pigus ir plačiai prieinamas ligos baigties prognozavimo rodiklis.

## Introduction

Thoracic empyema represents a life-threatening disease in both adults and children, requiring urgent diagnostic and treatment measures. Between 20% and 30% of patients either die or require additional surgical intervention in the first year after developing empyema [1]. Developments in the management in recent decades have not led to the desired survival rates and empyema is still associated with high morbidity and mortality [2], making early diagnosis and prognosis crucial for final outcome. Prediction of lethal outcome using routine laboratory markers is a rapid and inexpensive way to evaluate high-risk patients, enabling timely adjustment of treatment strategy.

Serum albumin and urea are easily assessable and low cost biomarkers, which are used widely as diagnostic and prognostic tools in everyday practice. It is thought that urea levels in peripheral blood can reliably reflect the association between renal status, nutritional status and metabolism [3,4]. Higher urea levels have been found to be a predictor of death in patients with thoracic empyema [2,5], critical illness [6] and sepsis [7]. Lower serum albumin concentrations represent an unfavorable prognostic factor, which is common in critically ill patients, especially in those with infections and sepsis, and is strongly associated with adverse outcome [8]. Hypoalbuminemia is a major cause of disorders in nutrient absorption, immune response, and production of plasma proteins [9]. Hypoalbuminemia also significantly affects the pharmacokinetics and pharmacodynamics of anti-infective therapy, which further complicates the disease severity [8]. Various studies have reported an association between lower albumin levels and mortality in patients with thoracic empyema [5,10,11], pneumonia [12], lung cancer [13], and sepsis [14,15].

The serum urea to albumin ratio (UAR), which represents an indirect indicator of the degree of dehydration, nutritional status and functional reserves of the liver and kidneys [16], has recently been found to be a significant predictor of death in pneumonia [17], critical illness [18,19], sepsis [20-22] and septic shock [23]. However, the ability of UAR to prognosticate outcome in thoracic empyema has not yet been evaluated. Therefore, we aimed to find an association between UAR and mortality in such clinical setting.

## Material and methods

This retrospective study was performed in a Clinic of Thoracic Surgery at a University Hospital on eighty-four patients with thoracic empyema over a 34-month period (January 2021 to November 2023). We reviewed the medical records of all adult patients with empyema thoracis who underwent emergency surgery. The diagnosis was determined by presence of pus or positive culture in pleural space [1]. Our exclusion criteria were age < 18 years and use of immunosuppressants, however, no patient met them and finally 84 participants were involved in the analysis.

Demographic, laboratory and clinical data were obtained from electronic medical records of each patient on admission to the clinic. The primary endpoint of the study was to evaluate the significance of UAR in predicting of fatal outcome, whereat in-hospital mortality was considered.

A presence of systemic inflammatory response syndrome (SIRS) was defined as two or more of the following four signs: a heart rate > 90/minute, a respiratory rate > 20/minute, a body temperature < 36°C or > 38°C and a white blood cell (WBC) count < 4x109/L or > 12x109/L [24]. A positive quick-sequential organ failure assessment (qSOFA) score was identified as ≥ 2 of the following 3 criteria: a systolic blood pressure ≤ 100 mmHg, a respiratory rate ≥ 22/minute and a Glasgow Coma Scale < 15 points [25].

The UAR was calculated by the equation: urea (mg/dL)/albumin (mg/dL) x 1000 [18] based on laboratory results at admission. Serum levels of urea and albumin were analyzed using a spectrophotometric method (AU480 Chemistry Analyzer, Beckman Coulter) in the hospital laboratory.

In all 84 patients WBC, SIRS and qSOFA were determined, while due to missing data in medical records we recorded serum C-reactive protein (CRP) and urea (Ur) in 81 patients, total protein (TP) in 75 patients, albumin (Alb) and UAR in 74 patients.

SPSS Statistics 19.0 (IBM, Chicago, Illinois, USA) was used for data analysis. Prognostic values of UAR, SIRS and qSOFA were compared performing area under receiver operating characteristics (AUROC) curves. Continuous variables were expressed as mean (±SD) or median (IQR) for normally or nonnormally distributed data, respectively. Categorical variables were presented as frequency (%) and compared by Fisher exact test or Chi-square test. A p-value was considered significant at < 0.05.

## Results

### Basic characteristics

Of the total of 84 patients, nine (10.7%) had a lethal outcome. Despite the observation that all patients who died were male, no significance was established for this variable according to outcome (p = 0.194). No significant differences between survivors (S) and nonsurvivors (NS) were established also for age (p = 0.186), hospital stay (p = 0.302), blood type (p = 0.092), comorbidity (p = 1.00), stage (p = 0.217) and location of empyema (p = 0.298). In contrast, patients who underwent minimally invasive surgery were found to have a higher chance of favorable outcome (p = 0.031) [Table 1].

**Table 1:** Basic characteristics

Variable	Total population	Survivors (75)	Non-survivors (9)	p-value
Age, years (IQR)	62.5 (47–70.5)	61 (45–69)	68 (58–74.5)	0.186
Sex, n (%)				
*male/female*	66(78.6)/18(21.4)	57(76)/18(24)	9(100)/0(0)	0.194
Hospital stay, days (IQR)	10 (7–14)	10 (8–14)	7 (3.5–18)	0.302
Blood type, n (%)				0.092
*A+*	30 (35.7)	29 (38.7)	1 (11.1)	
*A-*	7 (8.3)	5 (6.7)	2 (22.2)	
*B+*	9 (10.7)	6 (8)	3 (33.3)	
*B-*	2 (2.4)	2 (2.7)	0 (0)	
*0+*	27 (32.1)	25 (33.3)	2 (22.2)	
*0-*	3 (3.6)	3 (4)	0 (0)	
*AB+*	6 (7.1)	5 (6.7)	1 (11.1)	
*AB-*	0 (0)	0 (0)	0 (0)	
Location, n (%)				0.298
*Left hemithorax*	44 (52.4)	41 (54.7)	3 (33.3)	
*Right hemithorax*	40 (47.6)	34 (45.3)	6 (66.7)	
Stage, n (%)				0.217
*Exudative*	8 (9.5)	6 (8)	2 (22.2)	
*Fibrino-purulent*	70 (83.3)	64 (85.3)	6 (66.7)	
*Organizing*	6 (7.1)	5 (6.7)	1 (11.1)	
Surgical approach, n(%)				**0.031**
*Chest tube drainage*	21 (25)	18 (24)	3 (33.3)	
*VATS*	54 (64.3)	51 (68)	3 (33.3)	
*Thoracotomy*	9 (10.7)	6 (8)	3 (33.3)	
Comorbidity, n (%)	59 (70.2)	53 (70.7)	6 (66.7)	1.000
*Cardiovascular*	41 (48.8)	36 (48)	5 (55.6)	0.735
*Respiratory*	9 (10.7)	6 (8)	3 (33.3)	0.052
*Oncologic*	11 (13.1)	8 (10.7)	3 (33.3)	0.091
*Endocrine*	15 (17.9)	13 (17.3)	2 (22.2)	0.659
*Neurologic*	5 (6)	5 (6.7)	0 (0)	1.000
*Excretory*	5 (6)	5 (6.7)	0 (0)	1.000
*Digestive*	2 (2.4)	2 (2.7)	0 (0)	1.000
*Hematologic*	1 (1.2)	1 (1.3)	0 (0)	1.000

IQR – interquartile range; n – number of patients; % – percentages of patients; VATS – video-assisted thoracoscopic surgery.

### Laboratory parameters and prognostic scores

Laboratory parameters WBC (p = 0.452), CRP (p = 0.663), TP (p = 0.556) and Alb (p = 0.506) failed to discriminate patients who died from those who survived. In contrast, levels of serum urea were higher in NS (10.1 vs 5.85, p = 0.002). More than half of participants had clinical evidence of SIRS (52.4%), however no significance according to outcome was observed (NS = 77.8% vs S = 49.3%, p = 0.16). NS had higher median qSOFA score (1 vs 0, p = 0.003) and a positive qSOFA was found more frequent in these patients (NS = 44.4% vs S = 8%, p = 0.01). We found UAR as a significant unfavorable indicator, with median values approximately twice as high in patients with poor outcome (NS = 60.54 vs S = 35.34, p = 0.003) [Table 2].

**Table 2:** Laboratory parameters and prognostic scores

Variable	Total population	Survivors	Nonsurvivors	p-value
WBC, x109/L (IQR)	14.35 (10.48–22.7)	14.1 (10.47–20.7)	16.4 (11.02–25.04)	0.452
CRP, mg/L (IQR)	188 (107.7–299.5)	184.6 (112.2–285.7)	316.8 (74.6–338.6)	0.663
Ur, mmol/L (IQR)	6.4 (4.25–9.6)	5.85 (4.02–8.5)	10.1 (8.35–17.75)	**0.002**
TP, g/L ±SD	67.27 ± 9.15	67.49 ± 9.31	65.45 ± 8.02	0.556
Alb, g/L ±SD	29.78 ± 5.73	29.94 ± 5.88	28.5 ± 4.42	0.506
SIRS, points (IQR)	2 (1–2)	1 (1–2)	3 (1.5–3)	0.074
SIRS ≥2, n (%)	44 (52.4)	37 (49.3)	7 (77.8)	0.16
qSOFA, points (IQR)	0 (0–1)	0 (0–1)	1 (0–3)	**0.003**
qSOFA ≥2, n (%)	10 (11.9)	6 (8)	4 (44.4)	**0.01**
UAR, ratio (IQR)	37.15 (24.56–57.42)	35.34 (23.45–53.98)	60.54(51.99–105.38)	**0.003**
UAR > 51.1, n (%)	23 (31.1)	16 (24.2)	7 (87.5)	**0.001**

WBC – white blood cells; CRP – C-reactive protein; Ur – urea; TP – total protein; Alb – albumin; SIRS – systemic inflammatory response syndrome; qSOFA – quick-sequential organ failure assessment; UAR – urea to albumin ratio; IQR – interquartile range; SD – standard deviation; % - percentages of patients; n – number of patients.

### Sensitivity, specificity and AUROCs

**Figure 1:**
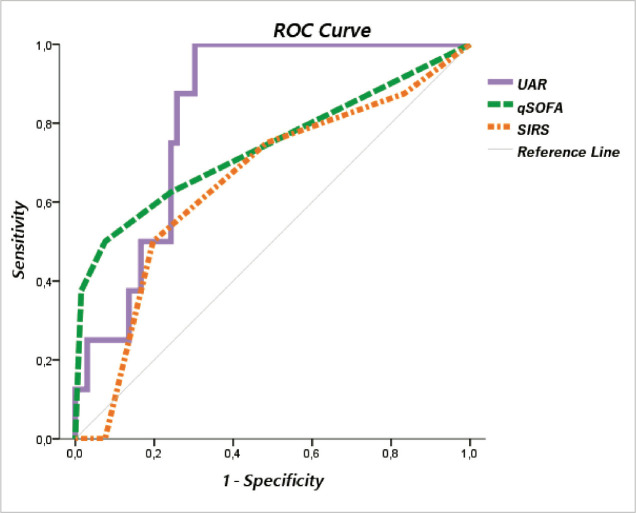
ROC Curves of UAR, qSOFA and SIRS in predicting mortality

The qSOFA showed higher accuracy to prognosticate mortality than SIRS (AUROC = 0.747, 95% confidence interval (CI) = 0.546–0.947 vs 0.676, 95% CI = 0.481–0.871). At a cut-off qSOFA ≥ 2 we observed a sensitivity of 44.4% and a specificity of 92%. ROC curve analysis revealed prognostic superiority of UAR in comparison to qSOFA and SIRS (AUROC = 0.828, p = 0.003 vs 0.747, p = 0.016 vs 0.676, p = 0.085) [Figure 1]. The established sensitivity and specificity for an optimal threshold UAR > 51.1 were 87.5% and 74.2%, respectively [Table 3].

**Table 3:** Sensitivity, specificity and AUROCs of SIRS, qSOFA and UAR

Variable	Cut-off	Sensitivity, %	Specificity, %	AUROC (95% CI)	p-value
SIRS	≥ 2	77.8	50.7	0.676 (0.481–0.871)	0.085
qSOFA	≥ 2	44.4	92	0.747 (0.546–0.947)	***0.016***
UAR	> 51.1	87.5	74.2	0.828 (0.725–0.930)	***0.003***

AUROC – area under receiver operating characteristics; CI – confidence interval (CI); SIRS – systemic in- flammatory response syndrome; qSOFA – quick-sequential organ failure assessment; UAR – urea to albumin ratio.

### Logistic regression

The ability of UAR to predict mortality was analyzed by direct logistic regression [Table 4]. The model using only the UAR was significant (p = 0.019), whereat the odds of adverse outcome increased by 1.019 by an increase on 1 point of the score.

**Table 4:** Direct logistic regression model for predicting lethal outcome

Variable	B	S.E.	Wald test	p-value	Odd Ratio (95% CI)
UAR	0.019	0.008	5.524	0.019	1.019 (1.003–1.035)

UAR – urea-to-albumin ratio.

## Discussion

Thoracic empyema affects over 65,000 patients annually in the United States and the United Kingdom [5] and is associated with significant healthcare costs and a major workload on hospital staff [26]. Approximately 1/4 of patients require prolonged hospitalization (more than 30 days) and between 10% and 20% of them had a poor outcome [27,28].

Despite the advances in management of empyema thoracis in recent decades, mortality rates remain unsatisfactory high. An early prognostic evaluation can provide an objective classification of the infection severity and differentiate high-risk patients in whom more aggressive therapeutic measures can be applied. Unfortunately, most patients with empyema do not seek help in time and are hospitalized with a significant delay, which further complicates effective treatment. Finding accurate predictive biomarkers that could contribute to early prognosis and early assessment of treatment regimen remains a matter of great importance.

On a global scale serum albumin and serum urea are among the most common laboratory parameters routinely used to evaluate nutritional balance and disease severity.

Higher serum urea was strongly associated with mortality in patients with thoracic empyema according to Rahman et al. [5] (p < 0.001) and Tsai et al. [2] (p = 0.049). In the present study, we confirmed these findings by observing that higher urea concentrations correlated with lethal outcome (p = 0.002). Conversely, Marks et al. [10] in thoracic empyema reported lack of prognostic significance (p = 0.055).

Hypoalbuminemia was also found as unfavorable factor in patients with thoracic empyema. Marks et al. [10] and Chu et al. [11] observed that patients who died had significantly lower albumin concentration (NS = 27 vs S = 32 g/L, p = 0.014 and NS = 25.1 vs S = 27.8, p = 0.035, respectively). Developing a prognostic score for thoracic empyema, Rahman et al. [5] reported that albumin levels > 27 mmol/L were indicative for increased chance of survival (p < 0.001). In contrast, we failed to establish an ability of serum albumin to differentiate patients at high risk of death (p = 0.506).

Determining the criteria with the greatest impact on the final outcome to be included in reliable prognostic score is not an easy task. A number of factors have already been proven to correlate with mortality in patients with empyema – advanced age, comorbidity, immunosuppression, prolonged hospital stay, sepsis, septic shock, multiple organ failure, poor control of the source of infection, nosocomial etiology [5,10,29]. A large number of researchers are still trying to deal with these problems, focusing on the prognostic capabilities of various scoring systems. However, most of the scores are complex and difficult for calculation, require multiple laboratory and clinical parameters, and are rarely used outside the intensive care units. This is not the case with simple laboratory prognostic indices or ratios, which are easily accessible and quickly calculated, requiring only 2 or 3 routine laboratory parameters. One of those ratios, the UAR has already shown promising predictive performance in various clinical settings [17-23], however it has never been investigated in thoracic empyema.

Our study showed a significant ability of UAR to discriminate patients according to final outcome, whereat nonsurvivors had almost two-fold higher values (NS = 60.54 vs S = 35.34, p = 0.003). Higher UAR in patients with poor outcome was observed also in critically ill by Rodrigues et al. [18] (NS = 23.7 vs S = 10.9, p < 0.001), in pneumonia by Ugajin et al. [17] (NS = 12.77 vs S = 5.26, p < 0.001), in sepsis by Min et al. [21] (NS = 10.29 vs S = 7.31, p < 0.001) and Han et al. [22] (NS=10.45 vs S=5.28, p < 0.001), and in septic shock by Pereira et al. [23] (NS=44.4 vs S=32.9, p = 0.003).

We identified a good performance of UAR to predict mortality (AUROC = 0.828) with sensitivity of 87.5% and specificity of 74.2%. In pneumonia, Ugajin et al. [17] reported a value similar to ours (AUROC = 0.830) with sensitivity and specificity of 57.9% and 94.5%. Lower prognostic value was found by Gundpatil et al. [19] (AUROC = 0.791, sensitivity = 67%, specificity = 83%) and Rodrigues et al. [18] (AUROC = 0.7201, sensitivity = 83.21%, specificity = 60.81%) in critical illness, by Han et al. [22] (AUROC = 0.741) and Wang et al. [20] (AUROC = 0.661) in sepsis, and by Pereira et al. [23] (AUROC = 0.617, sensitivity = 47.4%, specificity = 66.3%) in septic shock.

In the present study we demonstrated for the first time that UAR is an independent predictor of lethal outcome in patients with thoracic empyema (OR = 1.019, p = 0.019). UAR was also reported as independent factor for predicting death in pneumonia by Ugajin et al. [17] (OR = 1.10, p = 0.037), in sepsis by Han et al. [22] (OR = 1.056, p < 0.001) and Wang et al. [20] (OR = 1.032, p < 0.001) and in septic shock by Pereira et al. [23] (OR = 1.013, p = 0.007).

ROC Curve analysis in our study revealed prognostic superiority of UAR to qSOFA and SIRS (AUROC = 0.828 vs 0.747 vs 0.676), whereat it is the only factor with good ability to discriminate NS (AUROC of UAR is greater than 0.8). This is the first study (to the best of our knowledge) which investigated prognostic performance of UAR in patients with thoracic empyema and found a significant association with adverse outcome.

As limitations of our study we can highlight the single-center experience, the retrospective design and the small sample size.

## Conclusion

On the basis of our results we can conclude that the urea-to-albumin ratio represents a valuable marker for early prognosis in thoracic empyema, which can easily and inexpensively assess patients at high risk of lethal outcome

## References

[1] Garvia V, Paul M (2023). Empyema. In: StatPearls.

[2] Tsai YM, Gamper N, Huang TW, Lee SC, Chang AH (2019). Predictors and Clinical Outcomes in Empyema Thoracis Patients Presenting to the Emergency Department Undergoing Video-Assisted Thoracoscopic Surgery. J Clin Med.

[3] Kamar C, Ali A, Altun D (2017). Evaluation of risk factors and development of acute kidney injury in aneurysmal subarachnoid hemorrhage, head injury, and severe sepsis/septic shock patients during ICU treatment. Ulus Travma Acil Cerrahi Derg.

[4] Bellomo R, Kellum JA, Ronco C (2017). Acute kidney injury in sepsis. Intensive Care Med.

[5] Rahman NM, Kahan BC, Miller RF, Gleeson FV, Nunn AJ, Maskell NA (2014). A clinical score (RAPID) to identify those at risk for poor outcome at presentation in patients with pleural infection. Chest.

[6] Arihan O, Wernly B, Lichtenauer M (2018). Blood Urea Nitrogen (BUN) is independently associated with mortality in critically ill patients admitted to ICU. PLoS One.

[7] Li X, Li T, Wang J (2021). Higher blood urea nitrogen level is independently linked with the presence and severity of neonatal sepsis. Ann Med.

[8] Wiedermann CJ (2021). Hypoalbuminemia as Surrogate and Culprit of Infections. Int J Mol Sci.

[9] Gibbs J, Cull W, Henderson W, Daley J, Hur K, Khuri SF (1999). Preoperative serum albumin level as a predictor of operative mortality and morbidity: results from the National VA Surgical Risk Study. Arch Surg.

[10] Marks DJ, Fisk MD, Koo CY (2012). Thoracic empyema: a 12-year study from a UK tertiary cardiothoracic referral centre. PLoS One.

[11] Chu PY, Wu YC, Lin YL (2022). Surgical Treatment for Empyema Thoracis: Prognostic Role of Preoperative Transthoracic Echocardiography and Serum Calcium. J Pers Med.

[12] Oliva A, Borrazzo C, Mascellino MT (2021). CURB-65 plus hypoalbuminemia: a new score system for prediction of the in-hospital mortality risk in patients with SARS-CoV-2 pneumonia. Infez Med.

[13] Tewari N, Martin-Ucar AE, Black E (2007). Nutritional status affects long term survival after lobectomy for lung cancer. Lung Cancer.

[14] Saucedo-Moreno EM, Fernández-Rivera E, Ricárdez-García JA (2020). Hypoalbuminemia as a predictor of mortality in abdominal sepsis. Hipoalbuminemia como predictor de mortalidad en sepsis de origen abdominal. Cir Cir.

[15] Omiya K, Sato H, Sato T (2021). Albumin and fibrinogen kinetics in sepsis: A prospective observational study. Crit Care.

[16] Akahane J, Ushiki A, Kosaka M (2021). Blood urea nitrogen-to-serum albumin ratio and A-DROP are useful in assessing the severity of Pneumocystis pneumonia in patients without human immunodeficiency virus infection. J Infect Chemother.

[17] Ugajin M, Yamaki K, Iwamura N, Yagi T, Asano T (2012). Blood urea nitrogen to serum albumin ratio independently predicts mortality and severity of community-acquired pneumonia. Int J Gen Med.

[18] Rodrigues HCN, Silva ML, Mantovani MDS (2023). Higher urea-to-albumin ratio is associated with mortality risk in critically ill COVID-19 patients. Clin Nutr ESPEN.

[19] Gundpatil DB, Somani BL, Saha TK, Banerjee M (2014). Serum urea:albumin ratio as a prognostic marker in critical patients with non-chronic kidney disease. Indian J Clin Biochem.

[20] Wang Y, Gao S, Hong L (2023). Prognostic impact of blood urea nitrogen to albumin ratio on patients with sepsis: a retrospective cohort study. Sci Rep.

[21] Min J, Lu J, Zhong L, Yuan M, Xu Y (2022). The correlation study between blood urea nitrogen to serum albumin ratio and prognosis of patients with sepsis during hospitalization. BMC Anesthesiol.

[22] Han T, Cheng T, Liao Y (2022). Analysis of the Value of the Blood Urea Nitrogen to Albumin Ratio as a Predictor of Mortality in Patients with Sepsis. J Inflamm Res.

[23] Pereira AG, Costa NA, Gut AL (2021). Urea to albumin ratio is a predictor of mortality in patients with septic shock. Clin Nutr ESPEN.

[24] Bone RC, Balk RA, Cerra FB (1992). Definitions for sepsis and organ failure and guidelines for the use of innovative therapies in sepsis. Chest.

[25] Singer M, Deutschman CS, Seymour CW (2016). The Third International Consensus Definitions for Sepsis and Septic Shock (Sepsis-3). JAMA.

[26] Davies HE, Davies RJ, Davies CW, BTS Pleural Disease Guideline Group (2010). Management of pleural infection in adults: British Thoracic Society Pleural Disease Guideline 2010. Thorax.

[27] Farjah F, Symons RG, Krishnadasan B, Wood DE, Flum DR (2007). Management of pleural space infections: a population-based analysis. J Thorac Cardiovasc Surg.

[28] Grijalva CG, Zhu Y, Nuorti JP, Griffin MR (2011). Emergence of parapneumonic empyema in the USA. Thorax.

[29] McCormack DJ, Anderson JR (2017). Empyema thoracis. Surgery (Oxford).

